# Impact of DWI and ADC values in Ovarian-Adnexal Reporting and Data System (O-RADS) MRI score

**DOI:** 10.1007/s11547-023-01628-3

**Published:** 2023-04-25

**Authors:** Lucia Manganaro, Sandra Ciulla, Veronica Celli, Giada Ercolani, Roberta Ninkova, Valentina Miceli, Andrea Cozzi, Stefania Maria Rizzo, Isabelle Thomassin-Naggara, Carlo Catalano

**Affiliations:** 1 Department of Radiological, Oncological and Pathological Sciences, Sapienza University of Rome, Policlinico Umberto I, Viale del Policlinico 155, 00161 Rome, Italy; 2 Unit of Radiology, IRCCS Policlinico San Donato, Piazza Malan 2, 20097 San Donato Milanese, Italy; 3 Faculty of Biomedical Sciences, University of Italian Switzerland (USI), Via Buffi 13, 6900 Lugano, Switzerland; 4 Service of Radiology, Imaging Institute of Southern Switzerland, Clinica Di Radiologia EOC, 6900 Lugano, Switzerland; 5 Service de Radiologie, Hôpital Tenon, Assistance Publique-Hôpitaux de Paris, Sorbonne Université, Paris, France

**Keywords:** O-RADS, Magnetic resonance imaging, Ovarian carcinoma, Diffusion-weighted imaging (DWI)

## Abstract

**Purpose:**

Introduce DWI and quantitative ADC evaluation in O-RADS MRI system and observe how diagnostic performance changes. Assess its validity and reproducibility between readers with different experience in female pelvic imaging. Finally, evaluate any correlation between ADC value and histotype in malignant lesions.

**Materials and Methods:**

In total, 173 patients with 213 indeterminate adnexal masses (AMs) on ultrasound were subjected to MRI examination, from which 140 patients with 172 AMs were included in the final analysis. Standardised MRI sequences were used, including DWI and DCE sequences. Two readers, blinded to histopathological data, retrospectively classified AMs according to the O-RADS MRI scoring system. A quantitative analysis method was applied by placing a ROI on the ADC maps obtained from single-exponential DWI sequences. AMs considered benign (O-RADS MRI score 2) were excluded from the ADC analysis.

**Results:**

Excellent inter-reader agreement was found in the classification of lesions according to the O-RADS MRI score (*K* = 0.936; 95% CI). Two ROC curves were created to determine the optimal cut-off value for the ADC variable between O-RADS MRI categories 3–4 and 4–5, respectively, 1.411 × 10^–3^ mm^2^/sec and 0.849 × 10^–3^ mm^2^/sec. Based on these ADC values, 3/45 and 22/62 AMs were upgraded, respectively, to score 4 and 5, while 4/62 AMs were downgraded to score 3. ADC values correlated significantly with the ovarian carcinoma histotype (*p* value < 0.001).

**Conclusion:**

Our study demonstrates the prognostic potential of DWI and ADC values in the O-RADS MRI classification for better radiological standardisation and characterisation of AMs.

## Introduction

### Background

Ovarian carcinoma is the second most frequent gynaecological cancer in Western countries and is the first cause of death due to malignant neoplasia of the female genital tract [1].

Ultrasonography (US) is considered the first-line imaging approach for the evaluation of adnexal masses (AMs); however, between 18 and 31% of AMs remain indeterminate after ultrasound using the International Ovarian Tumour Analysis (IOTA) simple rules or other ultrasound scoring systems [2–4].

MRI (magnetic resonance imaging) plays a key role as a second-level method in the evaluation of indeterminate adnexal masses detected on US. Recently, the American College of Radiology (ACR) Ovarian-Adnexal Reporting and Data System (O-RADS) MRI committee published a lexicon and risk stratification system for adnexal lesions [5, 6]. O-RADS-MRI allows stratification of the risk of malignancy of adnexal masses based on lesion composition, signal intensity characteristics and solid tissue enhancement pattern.

O-RADS MRI system is based on morphological high-resolution T1 and T2 WI, dynamic contrast-enhanced (DCE) MRI series (temporal resolution ≤ 15 s) [7, 8] and time–intensity curve (TIC) [9]. TIC is obtained from the DCE-MRI series by placing two circular ROIs within the solid tissue in the adnexal lesion and at the level of outer myometrium and then processed by the perfusion analysis software to determine whether a low-risk, intermediate-risk or high-risk curve is present.

The main limitation remains related to the unfeasibility to obtain the enhancement curve, especially when a proper DCE MRI protocol is not performed, not allowing the correct classification into categories 3, 4 and 5. Additionally, TIC for intermediate and high risk cannot be evaluated in patients submitted to hysterectomy [9].

As well demonstrated, limitations in the applicability of TIC are also showed in breast imaging reporting data system (BI-RADS) MRI—due to the great heterogeneity of breast tumours and in nonmass tumours. Actually, a recent study showed that the type II curve (intermediate risk of malignancy) was present not only in malignant lesions (50%) but also in 29.3% of benign lesions [10]. The overlap of TIC patterns between benign and malignant diseases in clinical settings may occur, resulting in inaccurate diagnosis [11].

The primary objective of this study is to systematically introduce DWI and quantitative ADC evaluation in ORADS MRI and observe how diagnostic performance changes. The secondary objective is to evaluate the validity and reproducibility of the O-RADS MRI scoring system among readers with different experience in female pelvic imaging. Finally, the last objective is to assess whether there is a correlation between ADC value and histotype in lesions classified as malignant is reliable.

## Materials and methods

### Patients and study setting

The study was approved by the institutional review board, and informed consent was required for data analysis.

This is a retrospective single-centre cohort study conducted between January 2015 and June 2022 in the Radiology Department of Umberto I Hospital, Sapienza University of Rome, Italy.

We initially identified 173 patients consecutively with 213 adnexal masses indeterminate on ultrasound examination.

Inclusion criteria were: age > 18 years, standardized MRI examination with DWI and DCE sequences, subsequent surgery with histological examination or stability at follow-up imaging for at least 1 year.

Exclusion criteria were: age < 18 years (n*.* 2), no standard MRI examination (*n* 25), previous hysterectomy (*n*. 3), acute symptoms (*n*. 2) and no histopathological findings or follow-up < one year (n*.* 9).

The final cohort included 140 patients with 172 adnexal masses.

Patient enrolment, data collection and lesion classification according to ORADS-MRI score were retrospective.

### Magnetic resonance imaging

All MRI examinations were performed on a 3-T system (GE Discovery MR 750, GE Healthcare, Milwaukee, WI, USA) and on a 1.5-T system (MAGNETOM Avanto; Siemens Healthcare, Erlangen, Germany) using a 32-channel phased-array coil positioned on the lower abdomen.

Before the beginning of the examination, 20 mg of joscine N-butylbromide (Buscopan; Boehringer Ingelheim, Ingelheim, Germany) was injected intravenously to reduce motion artefacts caused by bowel peristalsis, if not contraindicated.

The standard MRI protocol included the following sequences, focusing on the lower abdomen from the pubic symphysis to the iliac crests: T2 fast spin-echo (FSE) weighted imaging (WI). On the sagittal, axial and coronal planes; axial T1 FSE WI with and without fat saturation (LAVA-Flex implementation of Dixon method), axial diffusion weighted images (DWI) with b-values of 0–1000 s/mm^2^ to obtain apparent diffusion coefficient (ADC) maps; dynamic T1weighted 3D gradient-echo with fat saturation in the axial plane during contrast uptake and delayed post-contrast T1-weighted 3D gradient echo with fat saturation in the axial plane.

Gadolinium chelate (gadoteric acid) was given at a dose of 0.2 mL per kilogram of body weight by using a power injector at a rate of 2 mL/sec, followed by 20 mL of normal saline to flush the tubing. Images were obtained sequentially at 2.4-s intervals beginning 10 s before the bolus injection, for a total of 320 s, Table 1.

**Table 1:** MR scanning parameters in detail

	TR/TE (ms)	FOV (mm)	NEX	Matrix size	Slice thickness (mm)	Intersection gap (mm)	B values (s/mm2)	FA (°)	Temporal resolution (s)
Axial, sagittal and coronal FSE T2WI	3411/121	320 × 320	2	320 × 224	4	1	–	–	–
Axial FSE T1WI (w/wo FS)	400/10	240 × 240	2	320 × 244	4	1	–	–	–
Axial DWI	2000/57	240 × 240	2	160 × 80	3,5	0	0–500-1000	–	–
3D-DCE T1WI *( gadoteric acid 0,2 ml/kg; 2 ml/sec)*	5/2	310 × 310	1	288 × 160	3	0	–	25	7

*MR *protocol;* TR*: repetition time;* TE*: echo time;* FOV*: field of view;* NEX*: number of excitations;* FA*: flip angle;* WI*: weighted imaging;* FSE*: fast spin-echo;* FS*: fat saturation;* DCE*: dynamic contrast enhanced (gadoteric acid, 0,2 ml/Kg; 2 ml/sec)

### Image analysis

All images were analysed independently by two radiologists (S.C. and V.M.) with 4 years and 1 year of experience in female pelvic imaging, respectively.

All readers, blind to clinical and histological data, retrospectively classified adnexal masses according to the six categories of the O-RADS MRI scoring system, published by Thomassin et al. in January 2020 [5].

O-RADS MRI risk stratification system has six classification categories: O-RADS MRI 0 (incomplete examination), O-RADS MRI 1 (normal ovaries), O-RADS MRI 2 (almost certainly benign), O-RADS MRI 3 (low risk), O-RADS MRI 4 (intermediate risk) and O-RADS MRI 5 (high risk).

According to previously published studies, the following MRI characteristics were analysed for each adnexal mass [12–15]: laterality, morphology (unilocular or multilocular), wall and septa (thin or irregular), content (fluid, solid, mixed), tissue characteristics (solid, adipose, fibrotic, blood), T2weighted signal intensity (SI) and DWI SI, free intraperitoneal fluid, peritoneal implants, time intensity curve (TIC) of the solid component. A TIC is created by placing a region of interest (ROI) in the most enhancing part of any solid tissue of the lesion and another on the external myometrium, trying to avoid the external myometrial vessels or fibroids, in accordance with O-RADS MRI system [16].

A gradual increase in the signal intensity of the solid tissue, without a well-defined “shoulder”, was defined as curve type 1. A moderate initial increase in the signal intensity of solid tissue relative to that of myometrium, followed by a plateau, was defined as curve type 2. An initial increase in the signal intensity of solid tissue that was steeper than that of myometrium was defined as curve type 3[17].

### ADC

Two radiologists (S.C. and L.M.) with 4 and 27 years of experience in female pelvic imaging, respectively, analysed ADC maps obtained from single-exponential DWI sequences on a post-processing workstation (AW VolumeShare 7, GE Healthcare, Milwaukee, WI, USA).

A tissue is considered benign if it is hyperintense in ADC and hypointense at b 1000, while it is malignant if it is hypointense in ADC and hyperintense at b 1000 [18].

Both radiologists independently drew a two-dimensional (2D) region of interest (ROI) on the ADC map. In particular, a circular ROI was placed in the slice containing the darkest part of the lesion, corresponding to the highest signal intensity at high b-values in the DWI, and correlates with the area of contrast enhancement on the post-contrast image. Moreover, T2-weighted and DCE images were used as anatomical reference.

The ROI was positioned by excluding areas of macroscopic necrosis, surrounding structures and areas with susceptibility artefacts. When lesions with multiple solid components were found, 4 to 6 ROIs were positioned on the targets and the ROI with the lowest ADC value was recorded.

Adipose tissue and blood components show low signal in ADC and are a common pitfalls in DWI. A combination of low signal at b1000 and low signal in ADC associated with markedly hypointense tissue in T2 weighted sequence (dark T2/dark DWI) is referred to benign lesions with fibrotic content [14]. Adnexal masses, without enhancing solid tissue, with adipose, haematic or fibrotic contents, are considered benign (O-RADS MRI score 2) and were excluded from the analysis of the ADC value.

### Reference standard

Histopathological diagnosis or imaging follow-up for at least 1 year was the reference standard. Histological diagnosis, which is considered the gold standard, was performed after complete surgical excision or after biopsy for inoperable lesions. Lesions were analysed by a pathologist blinded to MRI findings with more than 5 years of experience in female genital tumours and were classified as benign, borderline and malignant lesions according to the World Health Organization’s (WHO) International Classification of Diseases for Oncology (ICD-O) [19]. Malignant lesions were further classified into low grade and high grade based on the extent of cell anaplasia and the percentage of undifferentiated cells [20].

### Statistical analysis

Statistical analysis was performed using SPSS 25 (IBM SPSS statistics). To define the optimal cut-off for the ADC variable in predicting O-RADS categories, two different ROC curves were made; if the AUC was significant, the optimal cut-off was identified, to maximize the sum of sensitivity and specificity. ANOVA test and Bonferroni test were performed to detect differences in the ADC variable according to histotype (borderline tumours, low-grade serous carcinomas or high-grade serous carcinomas + ovarian carcinomas G3). Chi-squared test, Gamma Index and Cohen’s Kappa were calculated to measure the agreement between two readers. Each test was considered statistically significant if the p-value was lower than 0.05.

## Results

**Fig. 1:**
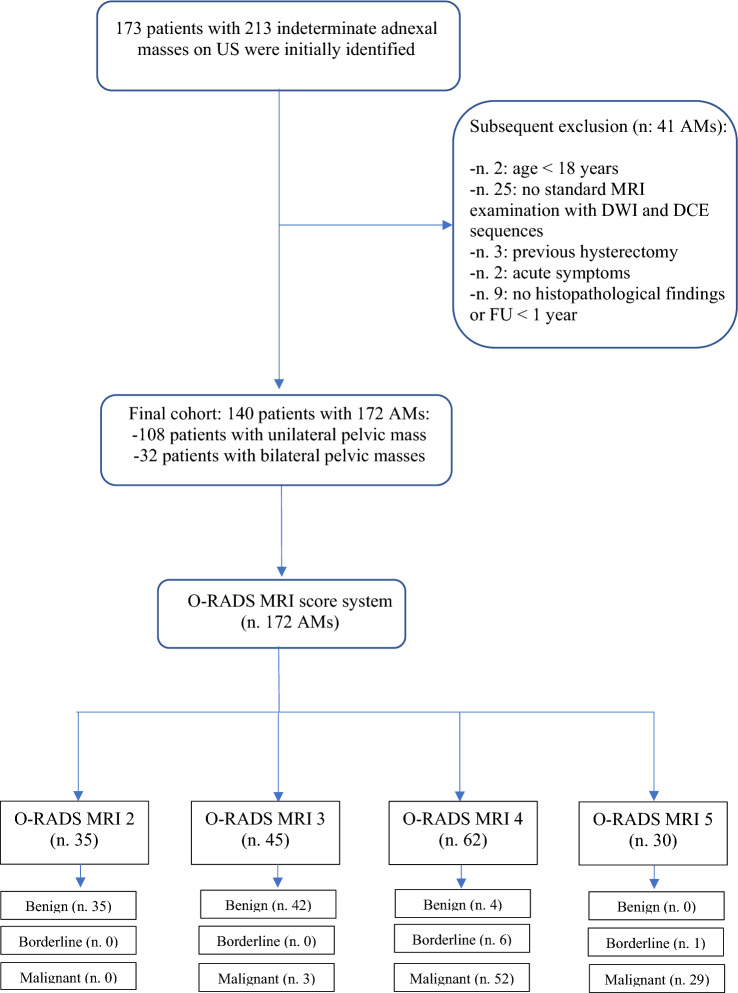
Flowchart of the study population. *US: ultrasound; AMs: adnexal masses; FU: follow-up; O-RADS: Ovarian-Adnexal Imaging Reporting and Data System; MRI: magnetic resonance imaging; DWI: diffusion-weighted imaging; DCE: dynamic contrast-enhanced*

A total of 173 women with 213 adnexal masses undetermined on ultrasound were subjected to MRI. In total, 41 adnexal masses were excluded; therefore, 140 patients with 172 ovarian masses were included in the final analysis. Among them, 108 had a single adnexal mass and 32 had bilateral adnexal masses. The flowchart of the study population is presented in Fig. 1. Of 172 lesions, 81 (47%) were benign; 91 (53%) malignant, among which the percentage of borderline tumours among all lesions considered to be malignant was 4% (7/172). Details of the histopathological findings are reported in Table 2. The malignancy rate included both malignant and borderline tumours. The malignancy rate was 0% (0/35), 6% (3/45), 93% (58/62) and 100% (30/30) in O-RADS MRI scores of 2, 3, 4 and 5, respectively.

**Table 2:** Histopathological findings of the 172 lesions according to the World Health Organization (WHO) Classification of Tumours (ICD-O)

Histopathological findings	*n*
Benign adnexal masses	81
Serous cystadenoma	11
Mucinous cystadenoma	6
Seromucinous cystadenoma	10
Serous cystadenofibroma	4
Mucinous cystadenofibroma	1
Mucinous adenofibroma	1
Mature teratoma	15
Endometriotic cyst	7
Brenner tumour	1
Fibroma and fibrothecoma	14
Tumour-like lesion *(functional cyst, dysfunctional cyst, corpus luteum, hydrosalpinx, haematosalpinx)*	11
Borderline adnexal masses	7
Serous borderline tumour	2
Mucinous borderline tumour	4
Serous borderline tumour with foci of intraepithelial carcinoma	1
Malignant adnexal masses	84
High-grade serous carcinoma	44
Low-grade serous carcinoma	8
Mucinous carcinoma	1
Clear cell carcinoma	2
Endometrioid carcinoma	5
Sex cord-stromal tumour	3
Germ cell tumour	1
Undifferentiated carcinoma	1
Fallopian tube carcinoma	2
Ovarian metastases	17

The mean age of these 140 women was 48.7 years (range: 18–83 years).

### Reproducibility and repeatability of the O-RADS MRI score

Excellent inter-reader agreement was found in the classification of lesions according to the O-RADS score between the two readers (*K* = 0.936; 95% CI). In fact, only 6/172 adnexal masses were classified differently between the readers (Table 3).

**Table 3:** Contingency table in the classification of lesions according to O-RADS MRI score between the two readers

	O-RADS Reader 1					Total
		2	3	4	5	
O-RADS Reader 2	2	33	2	0	0	35
	3	2	42	0	0	44
	4	0	1	59	0	60
	5	0	0	3	30	33
Total	35	45	62	30	172	

Frequency distributions of the O-RADS MRI score for AMs stratified by readers are presented in Table 4. The Chi-square test was statistically significant (p value < 0.001; 99% C.I), and there was strong dependence between the two classifications (Gamma Index = 0.999).

**Table 4:** Frequency distributions of O-RADS MRI scoring system for 172 adnexal masses stratified by reader

O-RADS MRI score	Risk category	Reader 1	Reader 2
2	Almost certainly benign	35 (20%)	35 (20%)
3	Low risk	45 (26%)	44 (25%)
4	Intermediate risk	62 (36%)	60 (35%)
5	High risk	30 (17%)	33 (19%)

### ADC

Two ROC curves were created to determine the optimal cut-off value for the ADC variable between O-RADS MRI categories 3–4 and 4–5. Thus, by obtaining two cut-off values of ADC between the different classes, it was possible, in some cases, to upgrade or downgrade O-RADS 3 and 4 AMs compared to the original O-RADS MRI classification. When an upgrading or downgrading of the lesion was not possible, the original O-RADS MRI score was confirmed. AMs originally classified as O-RADS MRI score 2 (*n*. 35) were confirmed in this category as they were all evaluated as benign, in agreement with the histological finding.

#### O-RADS MRI 3–4

**Fig. 2:**
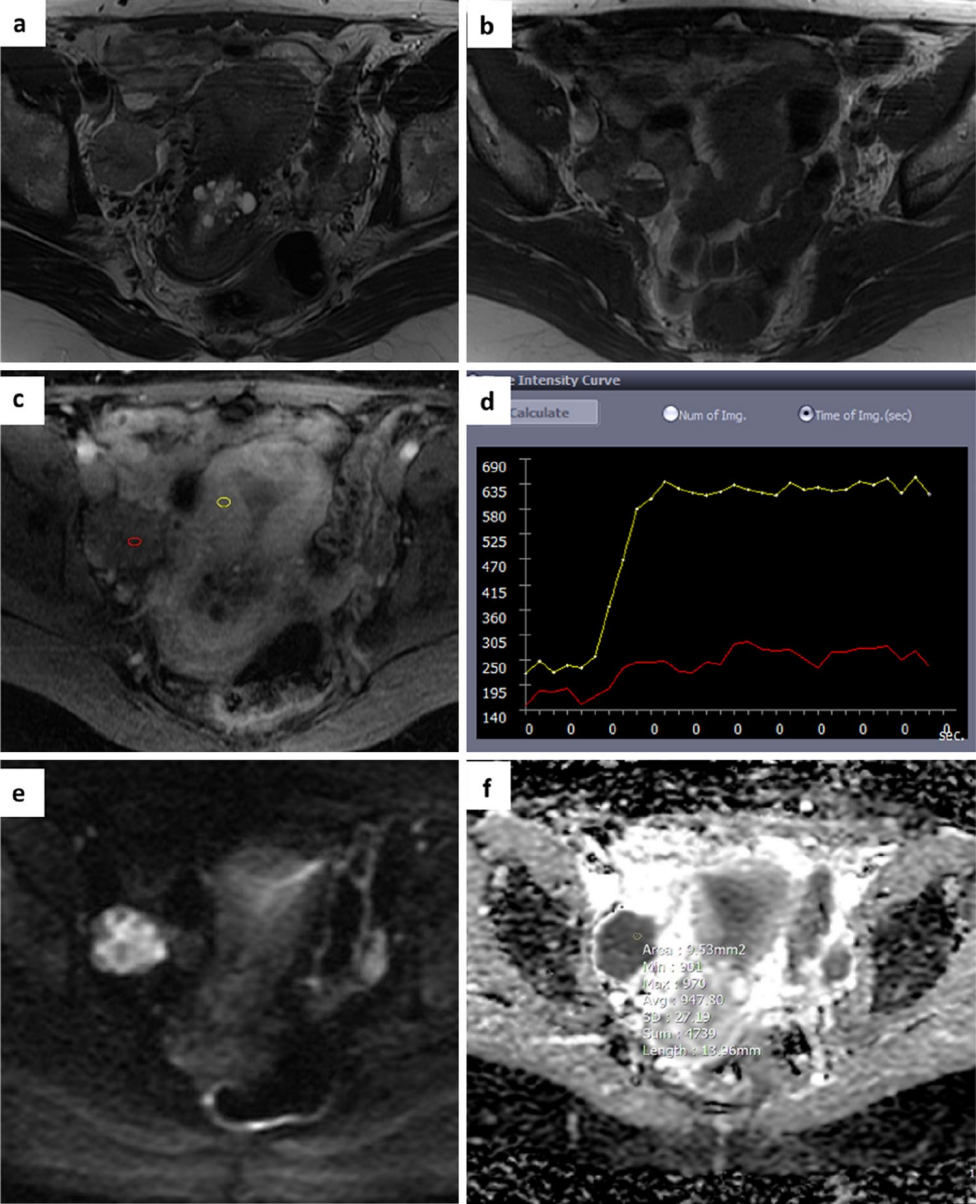
A 53-year-old woman. Axial T2 and T1-weighted images show a right adnexal mass with mixed content (solid, fluid and haematic-proteinaceous components) **a**,**b**. Perfusion-weighted sequence generates a low-risk curve (TIC type 1) **c**,**d** and an O-RADS MRI score 3 was attributed. DWI images and ADC map acquired in the axial plane show high signal restriction at b 1000 with ADC value of 0.947 × 10^–3^ mm^2^/sec **e**,**f**. Histology revealed a malignant adnexal lesion (high-grade serous carcinoma)

**Fig. 3:**
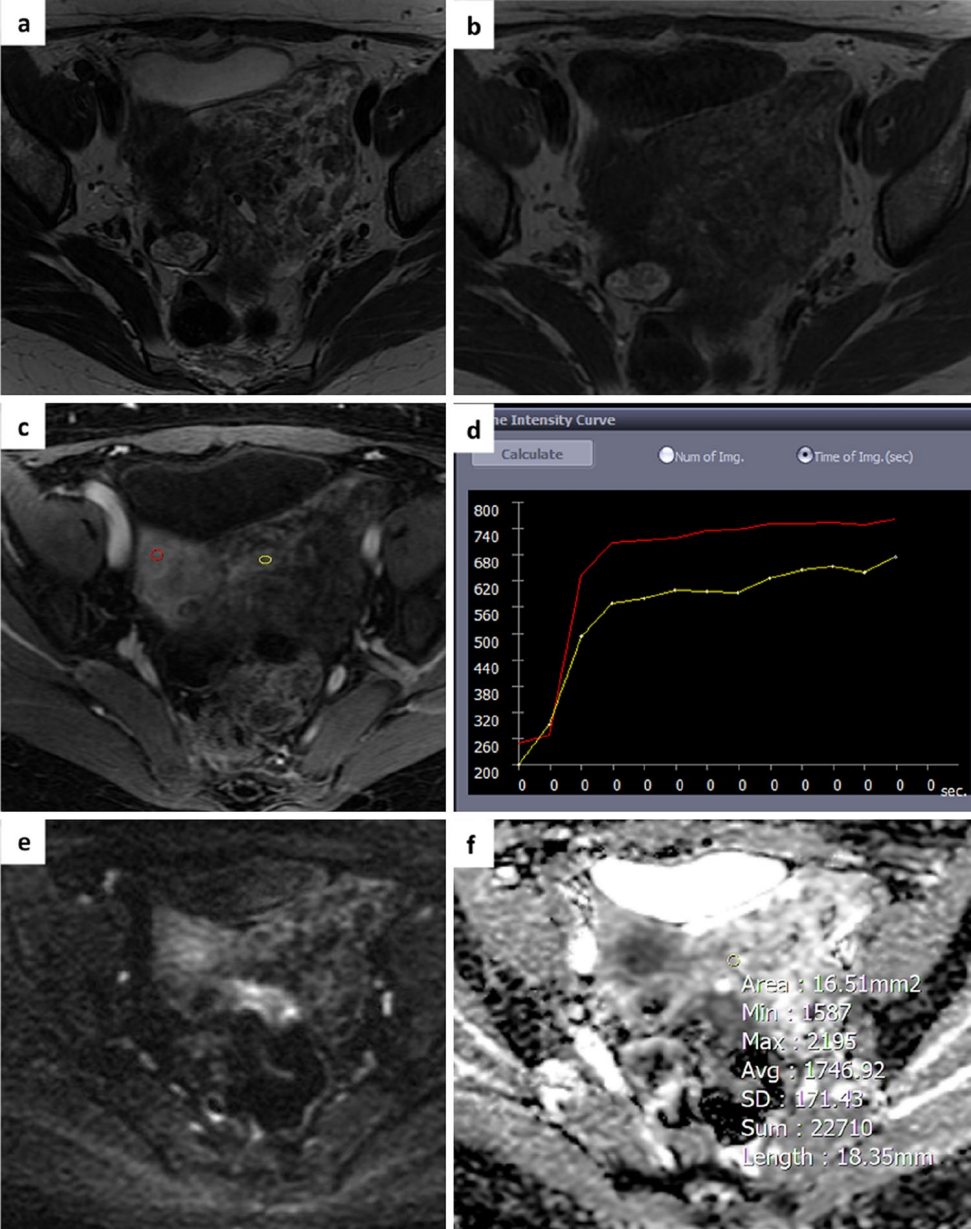
A 55-year-old woman with an indeterminate adnexal mass on ultrasound. Axial T2 and T1-weighted images show a voluminous mass in the left paramedian pelvis with mixed content due to the coexistence of solid components and fluid lacunae **a**,**b**. The perfusion sequence generates an intermediate risk curve (TIC type 2) **c**,**d** and an O-RADS MRI score 4 was attributed. DWI images and ADC map acquired in the axial plane show a slight restriction of the signal at b 1000 with an ADC value of 1.7 × 10^–3^ mm^2^/sec **e**,**f**. Histology revealed a benign lesion (ovarian fibroma)

The area under the curve (AUC) for O-RADS MRI score 3 and 4 was 0.951 (p value < 0.001) with an optimal ADC cut-off value of 1.411 × 10^–3^ mm^2^/sect. "Results"/45 AMs originally classified O-RADS MRI score 3 were upgraded to score 4 as they showed a ROI ADC < 1.411 × 10^–3^ mm^2^/sec. Histological data confirmed the malignancy of these lesions (a high-grade serous carcinoma, an ovarian metastasis from small cell lung carcinoma and a malignant germ cell neoplasm) (Fig. 2). Among 62 AMs classified O-RADS MRI score 4, 4 lesions with a ROI ADC > 1.411 × 10–3 mm2/sec were downgraded to score 3, in accordance with the histological finding of benignity (mucinous adenofibroma,, ovarian fibrothecoma, seromucinous cystadenoma, ovarian fibromatosis) (Fig. 3).

#### O-RADS MRI 4–5

All 30 adnexal lesions classified O-RADS MRI score 5, in relation to the presence of peritoneal implants or enhancing solid tissue with TIC type 3, remained in the same category with score 5. Histological findings confirmed the malignant nature of 29/30 adnexal lesions (21 serous adenocarcinoma, 2 fallopian tube carcinoma, 4 ovarian metastases, 1 undifferentiated carcinoma and 1 seromucinous adenocarcinoma). 1/30 mass was serous borderline tumour with foci of intraepithelial carcinoma.

**Fig. 4:**
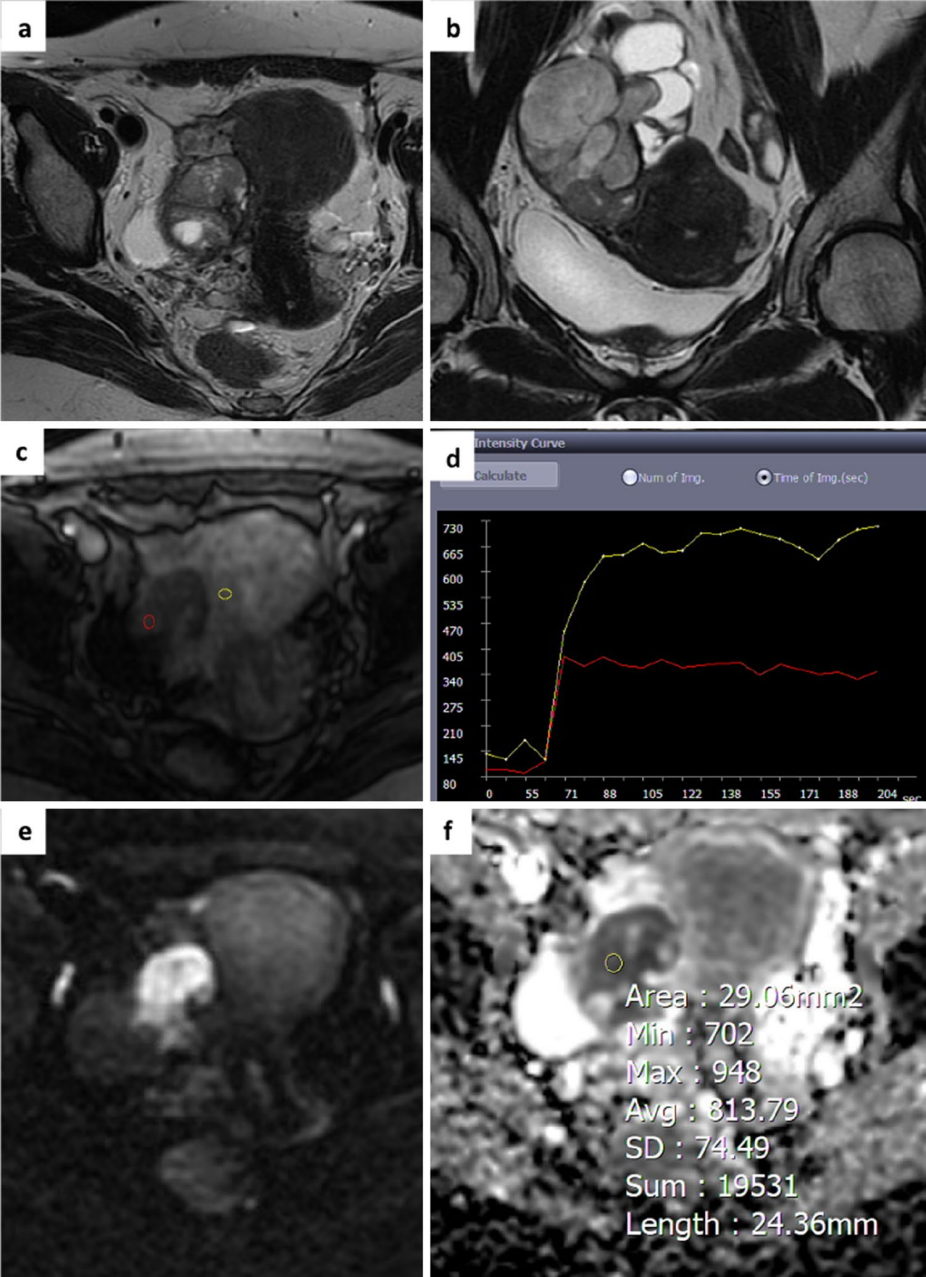
A 47-year-old woman. Axial and coronal T2-weighted images show a right adnexal mass with solid content and some fluid components **a**,**b**. The perfusion sequence generates an intermediate risk curve (TIC type 2) compared to the myometrium **c**,**d** and an O-RADS MRI score 4 was attributed DWI images and ADC map acquired in the axial plane shows high signal restriction at b 1000 with ADC value of 0.813 × 10^−3^ mm^2^/sec **e**,**f**. Histology revealed a malignant adnexal lesion (high-grade serous carcinoma).

The area under the curve (AUC) for O-RADS MRI score 4 and 5 was 0.630 (p value < 0.05) with an optimal ADC cut-off value of 0.849 × 10^–3^ mm^2^/sec. 22/62 AMs originally classified O-RADS MRI score 4 were upgraded to score 5 as they showed a ROI ADC < 0.849 × 10^–3^ mm^2^/sec. Histological data confirmed the malignancy of these lesions (13 high-grade serous adenocarcinomas, 2 ovarian metastases, 4 endometrioid adenocarcinomas, 3 sex cord-stromal tumour) (Fig. 4). Thirty-six out of 62 adnexal lesions, in accordance with TIC type 2, remained in O-RADS MRI score 4.

### Histotype and ADC

From a total of 91 lesions (84 malignant and 7 borderline AMs), 27 malignant tumours were excluded from the analysis: in detail, 14 ovarian metastasis and 13 adnexal lesions due to the lack of grading in the histopathological report. Overall, we evaluated 64 AMs (7 borderline and 57 malignant). In total, 57 malignant lesions included: 44 high-grade serous carcinomas (HGSC), 5 G3 ovarian carcinomas (1 germ cell tumour, 2 endometrioid carcinomas, 1 undifferentiated carcinoma and 1 sex cord-stromal tumour) and 8 low-grade serous carcinomas. We divided the adnexal masses into 3 groups: high grade (HGSC + G3), low grade (LGSC) and borderline. The aim of the analysis was to correlate the mean ADC value with the histotype.

The mean ADC values of the solid component of ovarian tumours were as follows: borderline 1.227 ± 0.17 (× 10^–3^ mm^2^ /s); low grade 1.068 ± 0.13 (× 10^–3^ mm^2^ /s); high grade 0.779 ± 0.11 (× 10^–3^ mm^2^ /s). Our results indicated a statistically significant difference in the mean ADC values between the borderline, low-grade and high-grade histotypes (*p* value < 0.001). In fact, the mean ADC values were statistically significant between borderline and low grade (*p* value: < 0.035, CI: 95%), between borderline and high grade (*p* value: < 0.001, CI 95%) and between low grade and high grade (*p* value: < 0.001, CI 95%).

## Discussion

Ovarian cancer is the fifth most common cancer death in women, accounting for more deaths than any other cancer of the female reproductive system.

O-RADS MRI scoring system represents the cornerstone of MRI classification of adnexal masses. In this study, we retrospectively analysed adnexal masses using the O-RADS MRI scoring system described by Thomassin-Naggara et al. [9].

O-RADS MRI scoring system has a high sensitivity and specificity (92% and 91%, respectively) for the evaluation of indeterminate adnexal masses on ultrasound, as reported in a recent meta-analysis [21] including 13 studies with 4520 adnexal lesions*.*

Our data are in agreement with the results of recently published studies that reported a sensitivity of the O-RADS MRI score between 85.6% and 93.5% and a specificity between 84.6% and 97.5% [5, 9, 22–29].

However, we found a discordance regarding the malignancy rate in the O-RADS MRI 4 score (about 93%) compared to other studies conducted previously by Basha (about 60%) [29], Thomassin et al. (about 60%) [5], Ruiz (about 57%) [22] and Sasaguri et al. (about 63%) [23]. Our study was conducted in a specialised tertiary centre, and this could explain how the high incidence of above average malignant lesions affected diagnostic performance, particularly in O-RADS MRI 4 category.

As reported by Rizzo et al. [21] MRI 4 assignments are related to the correct interpretation of TIC and the diagnostic performance is influenced by DCE MRI protocol, with a 90% summary specificity among studies where DCE sequences were performed with a 15 s or less temporal resolution (coefficient, 0.4; *P* = 0.049).

Furthermore, there may be other limits that contribute to the misclassification of adnexal masses: lack of perfusion curve analysis software and visual assessment, incorrect interpretation of the curve due to difficulty in recognizing a shoulder and a plateau between a type 1 and 2 curve, overestimation of the curve in pelvic inflammatory disease or underestimation in the presence of hypovascularised tumours [30].

In this context, DWI could be useful in order to reduce the risk of misclassification.

Previous studies have reported the adjunctive role of DWI and ADC values in the classification of ovarian lesions. Hottat recently focused his study on the additional value of DWI in O-RADS MRI in a population of 131 women with ovarian lesions. The author included 42 lesions in O-RADS score 4: 21 (50%) malignant and 21 (50%) benign. “ROI ADC” and “whole-lesion ADC histogram mean” were significantly higher in benign compared to malignant lesions. A threshold ROI ADC mean value was identified that allowed O-RADS 4 lesions to be stratified into a low-intermediate (> 1.7) and intermediate-high (< 1.7) malignancy risk group. The sensitivity and specificity for diagnosing malignancy with an ADNEX MRI score of 4 or more were 95.5% and 86.6%, respectively, using the classic scoring system, and 95.7% and 93.3%, respectively, using the modified scoring system. Additionally, the author subclassified O RADS MRI score 4 into two subcategories (4a and 4b) on the basis of ADC values [26].

In a prospective study, Elshetry et al. analysed 116 adnexal lesions. They reported optimal thresholds to predict malignant adnexal lesions O-RADS MRI score > 3 and ADC mean value ≤ 1.08 × 10^3^ mm^2^/s obtaining a reduction of false positives, a significant increase in the specificity (97.1%, *p* = 0.005), PPV (95.4%, *p* = 0.002) and PLR (33.1, *p* < 0.0001), and nonsignificant change in the AUC (0.953, *p* = 0.252) and sensitivity (93.3%, *p* = 0.467) [28].

In accordance with the reported studies we evaluated how the integration of DWI and ADC values to T2 and DCE morphological sequences can improve the characterisation of adnexal masses, since the O-RADS MRI classification only included DWI in category 2 (black T2/DWI black) indicative of benignity.

In fact, the introduction of two ADC cut-off values 1.411 × 10^–3^ mm^2^/sec and 0.849 × 10^–3^ mm^2^/sec for O-RADS categories 3–4 and 4–5, respectively, allowed reclassifying some adnexal masses originally located in categories 3 (low risk) and 4 (intermediate risk).

We found a discrepancy between the two scoring systems in 29/172 lesions (17%). In detail, 25 AMs were upgraded: 22 lesions moved from category 4 (intermediate risk) to 5 (high risk) and 3 lesions from category 3 (low risk) to 4 (intermediate risk). In contrast, 4 masses downgraded from category 4 (intermediate risk) to 3 (low risk). The new classification matched the histopathological data.

**Fig. 5:**
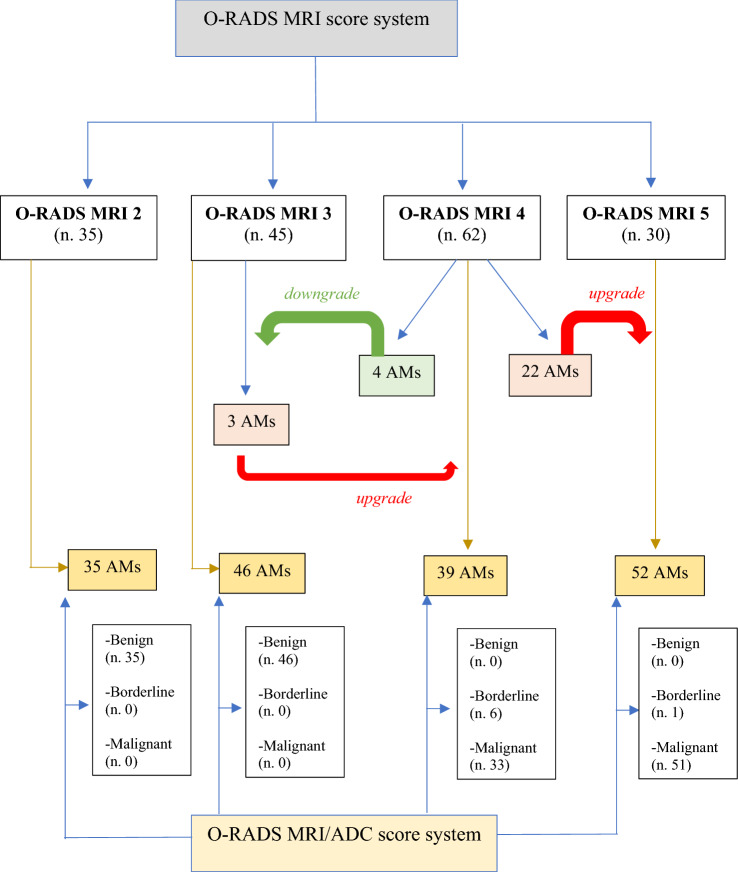
Flowchart of adnexal lesions in the new combined O-RADS MRI/ADC system *O-RADS: Ovarian-Adnexal Imaging Reporting and Data System; MRI: magnetic resonance imaging; AMs: adnexal masses; ADC: apparent diffusion coefficient.*

There was concordance between the two scoring systems in 143/172 (83%) adnexal masses. The new combined O-RADS MRI/ADC mean system reclassified adnexal masses as shown in Fig. 5.

The impact of DWI reduced the number of adnexal masses in category 4 (from 62 to 39) and increased it in category 5 (from 30 to 52), in agreement with the histopathological data.

We thus obtained an optimisation of the prevalence of malignancy in O-RADS 4 from 34 to 23% and O-RADS 5 from 17 to 30%. However, in category 3, the prevalence of malignancy was reduced from 2 to 0% (Fig. 2).

As reported in the literature, ADC values are correlated to the tumour cellularity; therefore, a low ADC value is associated with higher tumour cellularity, while a higher ADC value is associated with lower tumour cellularity. Thus, there is a strong correlation between the mean ADC value and histotype, as shown in other previous studies [31].

According to these data, we found the mean ADC values of borderline tumours were higher than those of low-grade serous carcinomas and high-grade carcinomas and were lower in high-grade serous carcinomas and G3 ovarian carcinomas, in agreement with other studies [32, 33].

Our study highlights as MRI system scoring could be improved by adding the ADC mean values reducing false positives and increasing specificity in O-RADS MRI 4 category.

Additionally, in patients where contrast agent cannot be administered (renal insufficiency, pregnancy) or where a time–intensity curve cannot be processed (hysterectomy or uterine agenesis) the feasibility of applying a biparametric study may help in the classification of indeterminate masses, as reported by Sahin et al.

They evaluated the performance of a noncontrast MRI protocol to characterise adnexal masses using exclusively T2-weighted sequences, DWI and ADC map, obtaining high sensitivity and specificity (respectively, 84.9% and 95.9% [34]).

Finally, our study confirms an excellent inter-reader agreement in the classification lesions according to the O-RADS score as just reported in the literature.

On the basis of our experience, the new modified O-RADS/ADC MRI score could allow a better diagnostic interpretation of adnexal masses enabling a tailored clinical and therapeutic management.

## Limitations

Our study has some limitations. Firstly, it was a single-centre retrospective study and a further studies with a larger cohort of patients are needed to standardise and validate our results.

Secondly, MRI examinations were performed by using two scanners operating at 1.5 T and 3.0 T, with the risk of obtaining inhomogeneous data on DCE and quantitative ADC values.

Thirdly we had a higher number of malignant lesions than in the other O-RADS MRI studies (53% vs 19%) [5].

Fourth, the evaluation was performed by readers with experience in gynaecological oncological imaging, which may create a limitation in the global standardisation of this score.

Finally, not all histological analyses of adnexal masses included tumour grading, which were not included in our research.

## Conclusion

O-RADS/ADC MRI score showed a better diagnostic performance than the classical O-RADS MRI system described by Thomassin-Naggara et al. [9]. We strongly believe that our study, although preliminary, demonstrates the important prognostic potential of DWI and ADC values in the O-RADS MRI classification system for better radiological standardisation and characterisation of adnexal masses. This system therefore improves the clinical and therapeutic management of patients and is essential to avoid unnecessary surgery in benign lesions and to improve the pharmacological and surgical management in malignant lesions.
